# Maternal plasma folate concentration is positively associated with serum total cholesterol and low-density lipoprotein across the three trimesters of pregnancy

**DOI:** 10.1038/s41598-020-77231-7

**Published:** 2020-11-19

**Authors:** Manoela T. da Silva, Maria F. Mujica-Coopman, Amanda C. C. Figueiredo, Daniela Hampel, Luna S. Vieira, Dayana R. Farias, Setareh Shahab-Ferdows, Lindsay H. Allen, Alex Brito, Yvonne Lamers, Gilberto Kac, Juliana S. Vaz

**Affiliations:** 1 Graduate Program in Food and Nutrition, Faculty of Nutrition, Universidade Federal de Pelotas, Rua Gomes Carneiro, 1, Pelotas, RS 96010-610 Brazil; 2 Food, Nutrition and Health Program, Faculty of Land and Food Systems, The University of British Columbia, Vancouver, BC Canada; 3 Nutritional Epidemiology Observatory, Josué de Castro Nutrition Institute, Department of Social and Applied Nutrition, Rio de Janeiro Federal University, Rio de Janeiro, Brazil; 4 USDA-ARS Western Human Nutrition Research Center, Department of Nutrition, University of California, Davis, CA USA; 5 Graduate Program in Epidemiology, Department of Social Medicine, Federal University of Pelotas, Pelotas, Brazil; 6 Laboratory of Pharmacokinetics and Metabolomic Analysis, Institute of Translational Medicine and Biotechnology, I.M. Sechenov First Moscow State Medical University, Moscow, Russia; 7 Department of Population Health, Nutrition and Health Research Group, Luxembourg Institute of Health, Strassen, Luxembourg

**Keywords:** Nutrition, Epidemiology

## Abstract

Increased first-trimester low-density lipoprotein (LDL-C) concentration has been associated with adverse pregnancy outcomes, such as gestational diabetes. The B vitamins folate, B-6, and total B-12 are key for the methyl group-dependent endogenous synthesis of phosphatidylcholine, which is needed for lipoprotein synthesis, e.g., very low-density lipoprotein (VLDL), the precursor of circulating LDL-C. Maternal B-vitamin concentration usually declines across trimesters. Whether changes in maternal B-vitamin concentrations are associated with total cholesterol (TC), triglycerides (TG), and lipoprotein concentrations is unknown. Therefore, we explored the association between plasma folate, vitamin B-6 in the form of pyridoxal 5′-phosphate (PLP), and total B-12 with serum TC, LDL-C, HDL-C, and TG concentrations across trimesters. This secondary analysis used data of a prospective pregnancy cohort study included apparently healthy adult women (*n* = 179) from Rio de Janeiro, Brazil. The biomarkers were measured in fasting blood samples collected at 5–13, 20–26, and 30–36 weeks of gestation. The associations between B vitamins and lipid concentrations across trimesters were explored using linear mixed-effect models. Among B vitamins, only plasma folate was positively associated with TC (β = 0.244, 95% CI 0.034–0.454) and LDL-C (β = 0.193, 95% CI 0.028–0.357) concentrations. The positive relationship of maternal folate and TC and LDL-C concentrations may indicate the importance of folate as a methyl donor for lipoprotein synthesis during pregnancy.

## Introduction

Folate, as a methyl donor, and vitamins B-6 (B-6) and B-12 (B-12), as cofactors, participate in one-carbon metabolism, which is vital for adequate cellular proliferation and differentiation^1^. Additionally, all these nutrients are important for the provision of methyl groups through the formation of the universal methyl donor, s-adenosyl methionine (SAM)^2,3^. SAM is required for the de novo synthesis of phosphatidylcholine (PC), the major phospholipid component of all circulating lipoproteins, from phosphatidylethanolamine (PE) by the action of the enzyme phosphatidylethanolamine N-methyltransferase (PEMT) in the liver^4,5^.

Circulating maternal concentrations of plasma folate, total B-12, and B-6, in the form of pyridoxal 5′-phosphate (PLP), decrease throughout gestation likely due to hemodilution, higher glomerular filtration rate, and increased mother-fetus nutrient transport^6,7^. Evidence from animal studies showed that mice fed a folate-deficient diet had significantly lower PC concentrations in the liver^8^, which we speculate may lead to lower hepatic synthesis of VLDL-C. Furthermore, it has been reported that the expression of lipoprotein-related genes in the liver significantly differs between mice that received a folate-deficient diet compared to those that received a control diet^9^, which may suggest a key role of folate in lipid metabolism through the provision of methyl groups for endogenous PC synthesis and regulating lipoprotein-related gene expression.

Evidence of the relationship between circulating folate, total B-12 and PLP concentrations and lipoprotein concentrations in humans is limited^10–12^. In healthy adults, plasma folate concentration has been positively associated with high-density lipoprotein cholesterol (HDL-C) concentration^12^, while an inverse association was found with low-density lipoprotein cholesterol (LDL-C) and total cholesterol (TC) concentrations^11,12^. Furthermore, a lower plasma B-12 concentration (< 220 pmol/L) was associated with a lower HDL-C concentration (< 40 mg/dL for healthy men and < 50 mg/dL for healthy women)^11^.

In pregnancy, an increase in circulating TC, LDL-C, HDL-C, fatty acids, and TG concentrations occurs across weeks of gestation to meet energy needs for maternal and fetal development^13–15^. However, high maternal lipoprotein concentrations, i.e., TG > 1.95 mmol/L and > 3.56 mmol/L and LDL-C > 3.27 mmol/L and 4.83 mmol/L in the first and second trimesters, respectively, have been associated with a higher risk of gestational diabetes in pregnant Chinese women^16^. Additionally, the results of a study in Dutch pregnant women showed that a TG concentration of 2.67 mmol/L was associated with a 7% increased risk of preeclampsia^17^. Despite the lack of an established cut-off for lipoprotein concentration across trimesters, these findings suggest that a potential excessive increment in maternal lipoprotein concentrations may increase the risk of adverse pregnancy outcomes.

Regardless of the suggested interrelationship between B vitamins and lipid metabolism, whether B vitamins are associated with lipoprotein concentrations during pregnancy is unknown. We have previously shown that changes in maternal PLP concentration were positively associated with docosahexaenoic acid (DHA) concentration and negatively associated with the n-6-to-n-3 fatty acid ratio across weeks of gestation^18^. However, the relationship between maternal plasma folate, B-6 and total B-12 concentrations and TC, TG, and lipoprotein concentrations throughout pregnancy has not yet been explored. Therefore, we performed a secondary analysis of data from a prospective pregnancy cohort study to investigate the association between folate, B-6 and total B-12 with serum lipid (TC, LDL-C, HDL-C, and TG) concentrations across trimesters in apparently healthy nonsupplemented women.

## Results

### Maternal characteristics

The mean age of women was 26.7 ± 5.6 years. Participants had an average of 8 years of education, and 37% were nulliparous. Most of the women (80%) were non-smokers, and 81% reported no alcohol consumption in the first trimester. According to the first trimester body mass index (BMI), 3% of women had underweight, 54% had normal weight, 31% and 12% had overweight and obesity, respectively. Ninety-four percent of women met the estimated average requirement (EAR) for B-12, while 70% and 91% did not meet the EAR for folate and B-6 for pregnant women, respectively (Table 1).

**Table 1:** First trimester characteristics of apparently healthy pregnant women at a public health center in Rio de Janeiro, Brazil, 2009–2012 (*n* = 179).

Characteristics	Mean (SD) or n (%)
Age, y	26.7 (5.6)
Education, y	9 (3)
**Parity, n (%)**	
0	66 (37)
≥ 1	113 (63)
**Smoking habit at first trimester, n (%)**	
Yes	35 (20)
No	144 (80)
**Alcohol intake at first trimester, n (%)**	
Yes	34 (19)
No	145 (81)
**First trimester BMI (kg/**m^2^**), n (%)**	
Underweight (< 18.5)	3 (1.7)
Normal weight (18.5–24.9)	97 (54.2)
Overweight (25–29.9)	56 (31.3)
Obese (≥ 30)	23 (12.8)
**Pre-pregnancy dietary intake**	
Folate intake, µg DFE/day	
≥ 520	54 (30)
< 520	125 (70)
B-6 intake, mg/day	
≥ 1.6	16 (9)
< 1.6	163 (91)
B-12 intake, µg/day	
≥ 2.2	168 (94)
< 2.2	11 (6)

Continuous variables are expressed as mean (SD); categorical variables are presented as *n* (%).

*SD* standard deviation, *BMI* body mass index, *DFE* dietary folate equivalents.

### Maternal B-vitamin, TC, LDL-C, HDL-C and TG concentrations across trimesters

**Figure 1:**
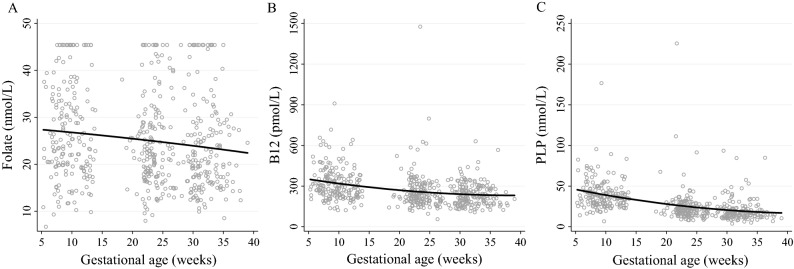
Maternal folate, total B-12 and PLP concentrations across trimesters in apparently healthy pregnant women followed at a public health center in Rio de Janeiro, Brazil, 2009–2012 (*n* = 179)^1^. Longitudinal linear regression coefficients (95% CIs) for weeks of gestation are as follows: (**A**) plasma folate: β =  − 0.11 (− 0.54, 0.33, *P* = 0.63; **B**); plasma total B-12: β =  − 7.86 (− 12.9, − 2.82, *P* < 0.05; (**C**); plasma PLP: β =  − 1.65 (− 2.48, − 0.82, *P* < 0.001). PLP, pyridoxal 5′-phosphate.

Per week of gestation, plasma PLP and total B-12 concentrations significantly decreased by 1.65 nmol/L and 7.86 pmol/L, respectively, while plasma folate concentration did not significantly change (Fig. 1). The median (IQR) plasma folate concentration only decreased between the first [24.8 (19.6; 33.97) nmol/L] and second trimesters [22.5 (17.9–29.0) nmol/L] (24.8 vs 22.5 nmol/L; *P* < 0.05), with no significant changes when comparing the second and third trimesters. Similarly, the total B-12 concentration significantly declined from the first to the second trimester (297.2 vs. 237.3 pmol/L), while no significant differences were found between the second and third trimesters. The PLP concentration decreased throughout all trimesters (36.3, 21.4, 16.8 nmol/L; *P* < 0.001 for all).

**Figure 2:**
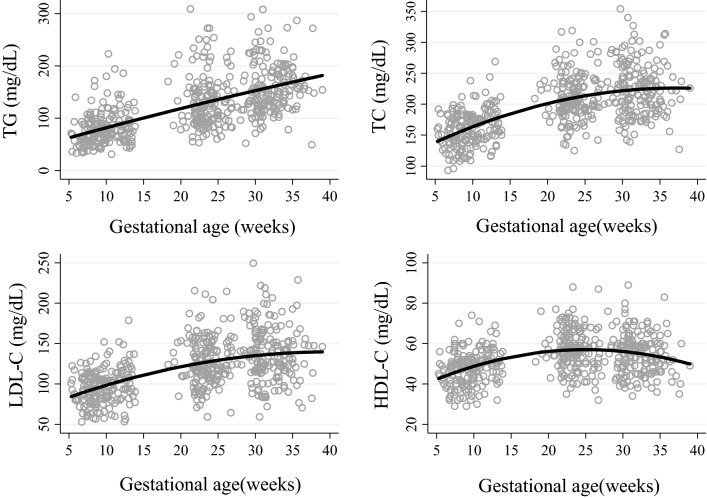
TG, TC, LDL-C, and HDL-C concentrations across trimesters in apparently healthy pregnant women followed at a public health center in Rio de Janeiro, Brazil, 2009–2012 (*n* = 181). Longitudinal linear regression coefficients (95% CIs) for weeks of gestation are as follows: TG: β = 4.01 (2.10, 5.33), *P* < 0.001; **B**) TC: β = 6.30 (5.33, 7.29), *P* < 0.001; **C**) LDL-C: β = 3.67 (2.90, 4.43), *P* < 0.001; HDL-C: β = 1.83 (1.52, 2.15), *P* < 0.001. *TG* triglycerides, *TC* total cholesterol, *LDL-C* low-density lipoprotein cholesterol, *HDL-C* high-density lipoprotein cholesterol.

Pregnant women showed a significant increase in all lipids per week of gestation: 4.0 mg/dL (TG), 6.3 mg/dL (TC), 3.7 mg/dL (LDL-C), and 1.8 mg/dL (HDL-C) (Fig. 2), and their concentrations also increased across trimesters (*P* < 0.05) (Table 2). In the first trimester, TC, LDL-C, and TG concentrations were significantly higher in pregnant women who were overweight or obese than in those who were underweight or normal weight (*P* < 0.05). In the second trimester, TG concentration was higher in smokers than in nonsmokers (*P* < 0.05), and in the second and third trimesters, older women (> 30 years) had higher TG concentrations than younger participants (< 30 years). The HDL-C concentration was lower in smokers than in nonsmokers in all trimesters (*P* < 0.05 for all) (Table 2).

**Table 2:** TC, LDL-C, HDL-C and TG concentrations stratified according to maternal characteristics across trimesters of apparently healthy pregnant women followed at a public health center in Rio de Janeiro, Brazil, 2009–2012 (*n* = 179)^.^

Variables	TC (mg/dL)			LDL-C (mg/dL)		
	1st trimester	2nd trimester	3rd trimester	1st trimester	2nd trimester	3rd trimester
	160.5 (28.9) [179] ^a^	210.3 (35.2) [168]^b^	224.2 (41.2) [172]^c^	96.7 (21.6) [179]^a^	127.1 (28.6) [168]^b^	137.6 (33.6) [172]^c^
**Age (years)**						
< 30	159 (28) [127]	210 (35) [118]	223 (41) [123]	97 (21) [127]	127 (29) [118]	138 (33) [123]
≥ 30	164 (31) [52]	212 (35) [50]	228 (41) [49]	97 (24) [52]	127 (29) [50]	138 (35) [49]
**Parity**						
0	161 (31) [66]	212 (36) [62]	226 (41) [64]	98 (24) [66]	129 (30) [62]	138 (36) [64]
≥ 1	160 (28) [113]	209 (35) [106]	223 (42) [108]	96 (20) [113]	126 (28) [106]	140 (32) [108]
**Smoking habit**						
Yes	153 (27) [11]	204 (31) [9]	208 (49) [9]	94 (21) [11]	119 (28) [9]	128 (34) [9]
No	161 (29) [168]	211 (35) [159]	225 (41) [163]	97 (22) [168]	128 (29) [159]	138 (34) [163]
**First trimester BMI**^a^						
Under/normal weight	156 (27) [100]^a^	209 (33) [91]	223 (41) [97]	94 (19) [100]^a^	128 (26) [91]	138 (31) [97]
Overweight/obese	166 (30) [79]^b^	211 (37) [77]	226 (42) [75]	101 (24) [79]^b^	126 (32) [77]	137 (37) [75]

	HDL-C (mg/dL)			TG (mg/dL)		
	1st trimester	2nd trimester	3rd trimester	1st trimester	2nd trimester	3rd trimester
	47.8 (8.5) [179]^a^	57.1 (9.8) [168]^b^	54.9 (10.0) [172]^c^	74 (57;94) [179]^a^	130.3 (48.1) [168]^b^	158.4 (48.7) [172]c
**Age (years)**						
< 30	47 (9) [127]	57 (9) [118]	55 (10) [123]	77 ± 31 (127)	125 ± 41 (118)^a^	152 ± 48 (123)^a^
≥ 30	49 (8) [52]	57 (11) [50]	55 (11) [49]	87 ± 34 (52)	144 ± 59 (50)^b^	174 ± 48 (49)^b^
**Parity**						
0	48 (9) [66]	58 (8) [62]	56 (9) [64]	76 ± 28 (66)	126 ± 46 (62)	161 ± 51 (64)
≥ 1	48 (8) [113]	55 (11) [106]	54 (11) [108]	82 ± 35 (113)	133 ± 49 (106)	157 ± 47 (108)
**Smoking habit**						
Yes	42 (8) [11]^a^	49 (9) [9]^a^	47 (8) [9]^a^	82 ± 21 (11)	181 ± 49 (9)^a^	166 ± 72 (9)
No	48 (8) [168]^b^	58 (10) [159]^b^	55 (10) [163]^b^	80 ± 33 (168)	128 ± 47 (159)^b^	158 ± 47 (163)
**First trimester BMI**^a^						
Under/normal weight	48 (9) [100]	57 (10) [91]	54 (10) [97]	74 ± 27 (100)^a^	122 ± 44 (91)^a^	151 ± 50 (97)^a^
Overweight/obese	48 (8) [79]	57 (10) [77]	57 (9) [75]	88 ± 37 (79)^b^	140 ± 51 (77)^b^	168 + 47 (75)^b^

Values are expressed as the mean (SD) [*n*].

^a^BMI, body mass index (kg/m^2^): under/normal weight < 25 kg/m^2^, overweight/obese ≥ 25 kg/m^2^; Labeled circulating TC, LDL-C, HDL-C and TG concentrations without a common superscript letter differ by maternal characteristics in each trimester, *P* < 0.05 (Student’s t-test) or between trimesters (ANOVA test and Bonferroni test as post hoc test).

*TC *total cholesterol, *LDL-C* low density lipoprotein cholesterol, *HDL-C* high density lipoprotein cholesterol, *TG* triglycerides.

### Maternal B-vitamin concentration and its association with TC, LDL-C, HDL-C, and TG concentration across weeks of gestation

In the adjusted longitudinal analysis, plasma PLP and total B-12 concentrations were not associated with TG, TC, HDL-C, and LDL-C concentrations, while plasma folate concentration was positively associated with TC and LDL-C concentrations across all trimesters (Table 3).

**Table 3:** Longitudinal associations between folate, PLP, and total B-12 concentrations and serum TC, LDL-C, HDL-C and TG concentrations across trimesters in apparently healthy pregnant women followed at a public health center in Rio de Janeiro, Brazil, 2009–2012.

Regression models	TC (mg/dL)		LDL-C (mg/dL)			HDL-C (mg/dL)			TG (mg/dL)	
	β^a^ (95% CI)	*P* ^b^	β^a^ (95% CI)		*P* ^b^	β^a^ (95% CI)		*P* ^b^	β^a^ (95% CI)	*P* ^b^
**Crude models**										
Folate (nmol/L)	0.252 (0.038, 0.466)	**0.021**	0.194 (0.027, 0.361)		**0.023**	0.018 (− 0.057, 0.093)		0.645	0.273 (− 0.012, 0.560)	0.061
PLP (nmol/L)	0.003 (− 0.120, 0.126)	0.958	− 0.003 (− 0.096, 0.091)		0.957	0.013 (− 0.028, 0.054)		0.525	− 0.005 (− 0.158, 0.148)	0.946
Total B-12 (pmol/L)	− 0.001 (− 0.020, 0.019)	0.957	− 0.005 (− 0.020, 0.010)		0.513	0.002 (− 0.005, 0.008)		0.589	0.019 (− 0.006, 0.043)	0.138
**Adjusted models**										
Folate (nmol/L)	0.244 (0.034, 0.454)	**0.023**	0.193 (0.028; 0.357)		**0.022**	0.007 (− 0.067, 0.081)		0.850	0.270 (− 0.011, 0.550)	0.060
PLP (nmol/L)	− 0.009 (− 0.131, 0.113)	0.890	− 0.011 (− 0.103, 0.081)		0.811	0.006 (− 0.035, 0.047)		0.767	0.009 (− 0.144, 0.161)	0.909
Total B-12 (pmol/L)	0.002 (− 0.018, 0.021)	0.890	− 0.004 (− 0.184, 0.011)		0.631	0.001 (− 0.005, 0.008)		0.652	0.022 (− 0.003, 0.046)	0.077

^a^Longitudinal linear regression coefficient.

^b^*P *value refers to the maximum likelihood estimator;* P* values were considered significant at < 0.05 are in bold. Folate model was adjusted for weeks of gestation, quadratic weeks of gestation, maternal age, parity, smoking, first trimester BMI and gestational dietary folate intake; PLP model was adjusted for weeks of gestation, quadratic weeks of gestation, maternal age, parity, smoking, first-trimester pregnancy BMI, gestational dietary B-6 intake; Total B-12 model was adjusted for weeks of gestation, quadratic weeks of gestation, maternal age, parity, smoking, first trimester BMI, gestational dietary B-12 intake.

*BMI* body mass index, *CI* confidence intervals, *TC* total cholesterol, *LDL-C* low density lipoprotein cholesterol, *HDL-C* high density lipoprotein cholesterol, *TG* triglycerides.

## Discussion

Folate, vitamin B-6, in the form of PLP, and total B-12 have interdependent roles in one-carbon metabolism, which is critical for the synthesis of the universal methyl donor SAM. Three SAMs are required for the endogenous synthesis of one PC by PEMT in the liver. An adequate synthesis of PC is needed for lipoprotein synthesis, such as VLDL-C^4^. To the best of our knowledge, this is the first study exploring the relationship between maternal folate, PLP and total B-12 with TC, TG and lipoprotein concentrations across trimesters. In this prospective pregnancy cohort study, folate concentration was positively associated with TC and LDL-C concentrations across trimesters.

Our results showed that maternal TC, LDL-C, HDL-C, and TG concentrations significantly increased between trimesters and weeks of gestation. Similar results have been reported in previous pregnancy studies^16,19,20^. Alterations in maternal lipid metabolism result from physiological changes to meet maternal and fetal needs for optimal gestation and lactogenesis^13^. In the early stage of pregnancy, there is a mobilization of fatty acids to storage in maternal adipose tissues, and the maternal body uses lipids for energy, characterized by an anabolic phase. Later, these reserves are gradually released to meet the growing fetal needs for essential lipids and energy, characterizing a catabolic phase^13,21^. The potential physiological mechanisms involved in the increase in TG, TC, and lipoprotein concentrations are likely related to pregnancy-related hormonal changes^13,22^. Specifically, it has been suggested that increasing estrogen concentration across trimesters promotes the production of VLDL-C in the liver, which can be further released into circulation. In circulation, VLDL-C can be converted to LDL-C^23^, which may explain the increasing LDL-C concentration across trimesters. Moreover, it has been reported that increasing estrogen concentration may decrease the expression and the lipolytic activity of hepatic lipase, which catalyzes the hydrolysis of TG^24^, therefore promoting TG accumulation in the liver, and it is subsequently used for VLDL-C synthesis. In contrast, estrogen^25^ and human placental lactogen hormone^26^ may promote the lipolytic activity in maternal adipose tissue which may additionally increase circulating lipoprotein and TC concentration.

Nonesterified fatty acids and glycerol released from maternal adipose tissues travel to the maternal liver, where they are re-esterified to TG and used for VLDL-C synthesis. Higher VLDL-C synthesis may lead to higher circulating LDL-C concentrations, which may also contribute to explaining the increased LDL-C that we found in late pregnancy.

In contrast to TC, LDL-C, HDL-C, and TG concentrations, maternal total B-12 and PLP concentrations significantly decreased across weeks of gestation, while no significant change was seen in folate concentration. Additionally, maternal folate and total B-12 concentrations declined between the first and second trimesters, whereas PLP concentration dropped across all trimesters. Similar results were found in pregnant Spanish women, in whom plasma folate and total B-12 concentrations decreased from 15 to 24–27 weeks of gestation^27^, and a significant decline in PLP concentration across trimesters was also reported in pregnant Canadian women^28^. Potential mechanisms involved in the decrease of the aforementioned B vitamins by trimester are likely related to increased mother-fetus transport, increased glomerular filtration and hemodilution due to the increment of blood volume during pregnancy^29–31^. Although lipids are also transported from the mother to the fetus, unlike B vitamins, endogenous production is mainly related to hormonal changes and pregnancy-related metabolic adaptations in the lipolytic activity of the maternal liver and adipose tissue, likely preventing the decline in lipoprotein concentrations across trimesters. Furthermore, pregnancy-related changes in the remethylation and transsulfuration rates of one-carbon metabolism^32^ (e.g., a higher transsulfuration rate and a higher remethylation rate in the first and third trimesters), which involve folate, total B-12, and PLP, may also partially explain the changes in circulating concentrations of these B vitamins across trimesters.

Our findings showed that maternal folate concentration was positively associated with LDL-C concentration across weeks of gestation. The potential mechanisms underlying this association may be related to the role of folate as a methyl donor for SAM formation. For example, a study in adults showed that adults in the highest serum folate quintile (folate concentration > 45 nmol/L) had significantly higher SAM concentration compared to those with serum folate concentration in the lowest quintile (< 27 nmol/L)^33^. SAM formation is critical for the endogenous synthesis of PC from PE by the action of the PEMT enzyme^34^. Synthesis of PC by PEMT enzyme accounts for ~ 30%, while the CDP-pathway accounts for ~ 70% of total PC synthesis in the liver^35^. PC is the major phospholipid component of lipoproteins and is required for VLDL-C, HDL-C, and cholesterol synthesis in the liver^36,37^. Despite that the CDP-pathway contributes the larger amount of PC, PEMT activity is upregulated in pregnancy due to the regulatory role of estrogen on PEMT expression^38^, which results in an increased PC and likely higher lipoprotein synthesis in late pregnancy^39^. Furthermore, mice receiving a folate-deficient diet showed lower PC concentrations in the liver, which may be potentially associated with lower VLDL-C synthesis and release to circulation^8^. After the hepatic release of VLDL-C into circulation, it can be converted to LDL-C after the removal of fatty acids by peripheral tissue^13^. Hence, increasing folate concentration may promote SAM synthesis and the subsequent endogenous synthesis of VLDL-C by the PC-PEMT pathway, which could lead to higher LDL-C concentrations.

We also found a positive association between plasma folate and TC concentrations across weeks of gestation. A recent animal study showed that male mice fed a supraphysiologic dose of folic acid, which is the synthetic form of folate, had reduced expression of cholesterol 7 alpha-hydroxylase (CYP7A1), a liver-specific enzyme that catalyzes the rate-limiting step of cholesterol conversion into bile acids^40^. The reduced CYP7A1 activity may result in increased intrahepatic cholesterol concentration. Additionally, higher LDL-C concentrations in the liver downregulate hepatic LDL-C receptor (LDLR)^41^, resulting in reduced liver uptake of LDL-C and therefore increased circulating LDL-C concentration, which may also contribute to explaining the positive association between folate and TC concentrations.

The strengths of this study included the prospective design and the availability of plasma and serum samples collected at each trimester, which allowed us to determine folate, PLP, total B-12, TC, LDL-C, HDL-C, and TG concentrations in all trimesters in apparently healthy pregnant women. Moreover, the use of linear mixed-effect (LME) models allowed us to estimate time-dependent changes in folate, PLP, total B-12, TC, LDL-C, HDL-C, and TG across weeks of gestation and to include pregnant women with missing biomarker data. However, our study also has a few limitations. Given that the present study is a secondary analysis of already collected maternal plasma and serum samples, we were unable to measure PC, PE, SAM or SAH concentrations. SAM production is one of the most important metabolic fates of dietary methionine intake^42^. However, we were unable to estimate methionine dietary intake. First, the food frequency questionnaire (FFQ) used in the present study was not designed to estimate methionine intake^43^. Second, the Brazilian table of food composition includes data on methionine content for less than 10% of the food items consumed by the participants. Additionally, the use of international databases was not an adequate strategy to estimate methionine, because the alternative food composition databases also lack data on methionine content. As a consequence, the estimation of dietary methionine intake would be not accurate. Furthermore, losses during follow-up affected the sample size over time and may have reduced the power to detect significant associations between maternal B vitamins, TC, lipoproteins, and TG concentrations.

In summary, our study showed that maternal plasma folate concentration was positively associated with serum TC and LDL-C concentrations across weeks of gestation. Our findings suggest that folate may play a key role in maternal lipoprotein status. Further studies are needed to understand the mechanisms underlying this association.

## Methods

### Study design and participants

This was a prospective pregnancy cohort study conducted in the city of Rio de Janeiro, Brazil. Pregnant women were enrolled from November 2009 to October 2011 and followed up across all trimesters: 5–13 weeks of gestation (first), 20–26 weeks of gestation (second), and 30–36 weeks of gestation (third). At each trimester, a study visit was scheduled during appointment days at a prenatal care center of the Brazilian Unified Health System, and blood samples were collected. Socioeconomic, demographic, reproductive and lifestyle information was collected at the first study visit using structured questionnaires administered by trained research assistants.

**Figure 3:**
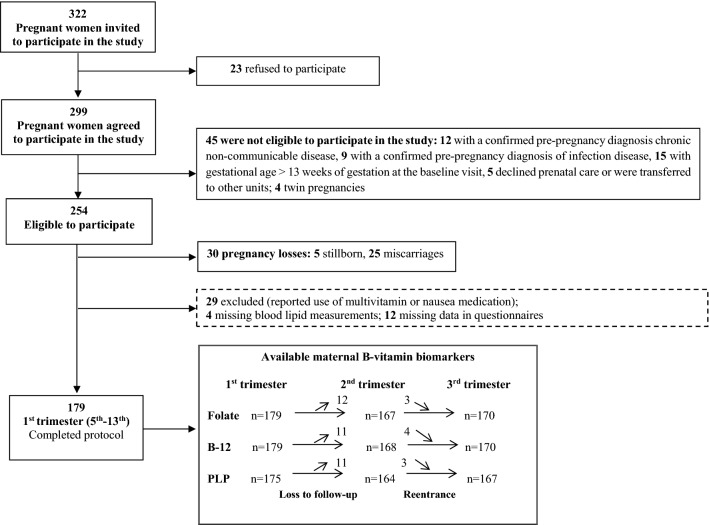
Flow chart of the study.

Pregnant women were included in the study if they met the following criteria at the screening: ≤ 13 weeks of gestation, aged 20–40 years, absence of diagnosed chronic diseases (except for obesity; BMI ≥ 30 kg/m^2^) and infectious diseases, singleton pregnancy, and being resident in the area covered by primary health center. In total, 322 pregnant women met the eligibility criteria and were invited to participate in the study; 23 refused to participate, and another 75 were lost after screening. Thus, the first-trimester sample comprised 179 pregnant women with available plasma samples for folate and total B-12 status assessment and 175 for PLP concentration (Fig. 3).

This study was registered in ClinicalTrials.gov (ID: NCT01660165) on 24 May 2012. The study protocol was approved by the Research Ethics Committee of the Institute of Psychiatry, Rio de Janeiro Federal University (Protocol #0012.0.249.000-09) and the Rio de Janeiro Municipal Secretary of Health (Protocol #0139.0.314.000-09). Written informed consent was obtained from all participants after all necessary clarifications had been provided before the study began. All ethical procedures of this study involving human beings followed the ethical standards laid down in the 1964 Declaration of Helsinki and its later amendments and Brazilian Resolution 196/96.

### Biochemical analyses

Blood samples (5 mL) were collected at enrollment and at each follow-up study visit after a 12-h overnight fast. Blood was collected in tubes with ethylenediamine tetra-acetic acid (EDTA) or separator gel and centrifuged at 5031 × 3*g* for 5 min at 21 °C within 1 h of blood collection. Serum and EDTA-treated plasma were separated into aliquots and stored at −80 °C until analysis.

Plasma folate and plasma total B-12 concentrations were determined by commercial automated competitive protein binding assays using chemiluminescence detection on the Cobas e411 bioanalyzer (Roche, Mannheim, Germany). B-6 was measured in plasma as the coenzymatic PLP form after derivatization to its semicarbazide derivative using HPLC with fluorescence detection^44^.

Serum TC, HDL-C and TG concentrations were determined at the Faculty of Pharmacy Clinical Analysis Laboratory (Federal University of Rio de Janeiro) using an automated analyzer (Labmax Plenno, Labtest Diagnostica, Minas Gerais, Brazil) and commercial enzymatic colorimetric assay kits (Labtest Diagnostica, Minas Gerais, Brazil). LDL-C (mg/dL) concentration was calculated as follows: TC—HDL-C—(TG/5)^45^.

### Obstetric, demographic and lifestyle data collection

Weeks of gestation were determined via ultrasound before 24 weeks of gestation (*n* = 158, 88%). When this information was not available in the obstetric record, gestational age was calculated based on the reported date of the last menstrual period (*n* = 21, 12%). Data about maternal age (< 30, ≥ 30), monthly per capita family income (Brazilian Reais, R$), education (years), parity (0, ≥ 1), leisure time physical activity (yes/no), smoking habit (yes/no), and alcohol consumption (yes/no) were collected at the first study visit.

### Maternal anthropometric measurements

Maternal body weight was measured to the nearest 0.1 kg using an electronic scale (Filizzola Ltd., São Paulo, Brazil). Maternal height (m) was measured in duplicate using a portable stadiometer to the nearest 0.1 cm (Seca Ltd., Hamburg, Germany). The anthropometric measurements were obtained according to standardized procedures and recorded by trained interviewers in the first study visit^46^. First trimester BMI was calculated using the formula: weight [kg]/(height [m^2^] and classified as underweight (< 18.5), normal weight (18.5–24.9), overweight (25.0–29.9), obese (≥ 30.0)]^46^. Since a small number of underweight cases were observed, in some descriptive analyses, we combined under- and normal weight and overweight and obese categories. Gestational weight gain (kg) was calculated as the difference between the last weight measured before delivery and the first trimester weight.

### Maternal dietary intake

Dietary intake was assessed at two time points: (1) between 5 and 13 weeks of gestation, at the first study visit, to assess the dietary intake during the 6 months preceding pregnancy (*prepregnancy dietary intake*), and (2) between 30 and 36 weeks of gestation to assess dietary intake during pregnancy (*gestational dietary intake*). A semi-quantitative FFQ was used to estimate usual daily dietary intakes of total fat, protein, carbohydrate, energy, folate, B-12 and B-6. The FFQ consisted of 81 food items, including non-alcoholic and alcoholic beverages. The FFQ has been previously validated for energy intake using the doubly labelled water method in the adult population of Rio de Janeiro^47,48^. Daily nutrient intake was calculated using DietSys version 4.01 software (National Cancer Institute, Bethesda, MD, USA). The nutrient database was constructed primarily with the nutritional composition of Brazilian food items^49^, and it was complemented with the database developed by the U.S. Department of Agriculture (USDA)^50^. The cut-offs used to classify dietary vitamin intake were based on the EAR for pregnant women, i.e., < 520/ ≥ 520 µg/day dietary folate equivalents for folate, < 2.2/ ≥ 2.2 µg/day for B-12 and < 1.6/ ≥ 1.6 mg/day for B-6^51^.

### Statistical analyses

#### Descriptive analysis

The distribution of the variables was assessed using visual inspection and the Shapiro–Wilk test. Maternal characteristics were described as the means and standard deviations (SD) for continuous variables and as absolute and relative frequencies (%) for categorical variables. We tested the differences in TC, TG, HDL-C, and LDL-C concentrations between trimesters using ANOVA and Bonferroni’s post hoc test. We further compared TG, TC, HDL-C and LDL-C concentrations at each trimester by first-trimester maternal characteristics (i.e., maternal age (< 30/ ≥ 30 years), parity (0, ≥ 1), smoking (yes, no), BMI (i.e., under/normal weight, overweight/obese) using Student's t test. Plasma folate, PLP, and total B-12 concentrations were compared by trimesters using a Kruskal–Wallis test and Wilcoxon rank-sum test as post hoc test.

#### Longitudinal analysis

The relationship between maternal folate, PLP, total B-12, TC, LDL-C, HDL-C, and TG concentrations and weeks of gestation was assessed using LME regression models and was illustrated using scatter plots containing longitudinal prediction and 95% confidence intervals. LME regression models were also used to assess the association between (1) maternal folate (folate model), (2) maternal PLP concentration (PLP model), and (3) maternal total B-12 concentration (total B-12 model) as exposure variables and TC, TG, HDL-C or LDL-C concentrations separately as outcome variables across weeks of gestation. LME models were fitted using weeks of gestation as random effects and using an unstructured covariance matrix to accommodate the variability in slopes over time among subjects, while all the other variables were included as fixed effects only. All models were further adjusted by the quadratic weeks of gestation variable to fit for the nonlinear variation of maternal biomarker concentration (e.g., plasma folate) across trimesters. LME models provide a combined estimate of between- and within-subject effects and accommodate time-dependent and time-independent covariates and allow measurements with unbalanced time intervals^52^.

Potential confounding factors were selected based on a direct acyclic graph (DAG). The DAG is a theoretical graphical model in which potential confounding factors that can distort the causal inference process are considered, allowing the identification of the minimum but sufficient set of covariates to remove confounding factors from the statistical analysis and help avoid inappropriate adjustments^53–55^. We created three DAGs to represent the association of (1) folate concentration, (2) PLP concentration, and (3) total B-12 concentration with TC, LDL-C, HDL-C, and TG concentrations, separately (Supplementary Figures S1, S2, S3), based on scientific evidence and biological plausibility of the association. To build the theoretical models, we considered all possible variables that were relevant to the association of B-vitamins and lipoprotein concentrations, even those not measured in the present study (unobserved or latent variables), such as methylene tetrahydrofolate reductase (MTHFR), cholesterol 7 alpha-hydroxylase (CYP7A1), 3-hydroxy-3-methyl-glutaryl-CoA reductase (HMG-COA reductase). The inclusion of latent variables is important to evaluate possible bias from unmeasured variables. Based on the results of the DAG, the following variables were included in the adjusted model: (1) folate model: weeks of gestation, quadratic weeks of gestation, maternal age, parity, smoking, first trimester BMI and gestational dietary folate intake; (2) PLP model: weeks of gestation, quadratic weeks of gestation, maternal age, parity, smoking, first-trimester pregnancy BMI, gestational dietary B-6 intake; and (3) total B-12 model: weeks of gestation, quadratic weeks of gestation, maternal age, parity, smoking, first trimester BMI, gestational dietary B-12 intake.

All statistical analyses were performed using STATA version 15 (Statacorp LLC, College Station TX, USA). The results were considered significant at P < 0.05.

## Supplementary information

Supplementary Figures.

## Data Availability

The dataset supporting the conclusions of this article is not openly available due to confidentiality of information. Consent for publication of raw data was not obtained, but a fully anonymous dataset can be obtained with the corresponding author.
