# Supplementary material for: Maternal plasma folate concentration is positively associated with serum total cholesterol and low-density lipoprotein across the three trimesters of pregnancy
Source: Sci Rep. 2020 Nov 19;10:20141. doi: 10.1038/s41598-020-77231-7 (PMC7677547; doi:10.1038/s41598-020-77231-7)
Supplement: Supplementary file 1 — Supplementary Figures. [file 41598_2020_77231_MOESM1_ESM.docx]

**Maternal plasma folate concentration is positively associated with serum total cholesterol and low-density lipoprotein across the three trimesters of pregnancy**

Manoela T da Silva, Maria F Mujica-Coopman, Amanda CC Figueiredo, Daniela Hampel, Luna S Vieira, Dayana R Farias, Setareh Shahab-Ferdows, Lindsay H Allen, Alex Brito, Yvonne Lamers_,_ Gilberto Kac^,^ Juliana S Vaz

**Supplementary figures**

**
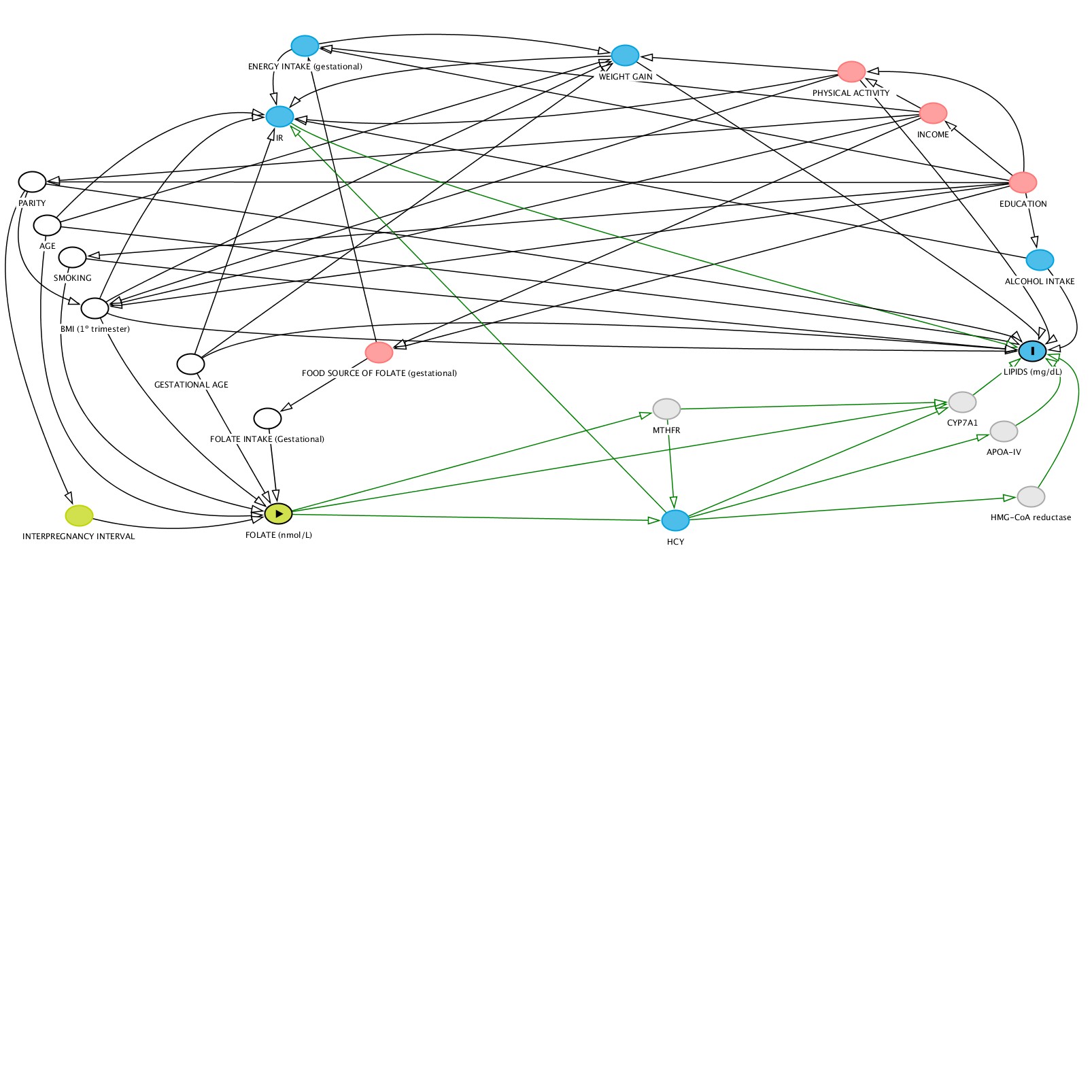
Supplementary figure 1**. Acyclic Directed Graph (DAG) representing the hypothesis of relations between plasma folate, serum lipids, and covariates.

Abbreviations: BMI, body mass index; IR, insulin resistance; CYP7A1, Cholesterol 7 alpha-hydroxylase; MTHFR, methylenetetrahydrofolate reductase; HCY, homocysteine; APOA-IV, apoprotein A-IV


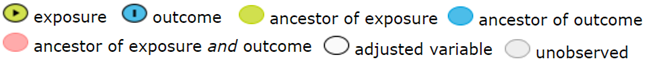


Drawn in DAGitty v 3.0

The reproducible DAG that was used to assess necessary adjustment sets (see Methods) can be found at <http://dagitty.net/dags.html?id=W0y4ni>

**
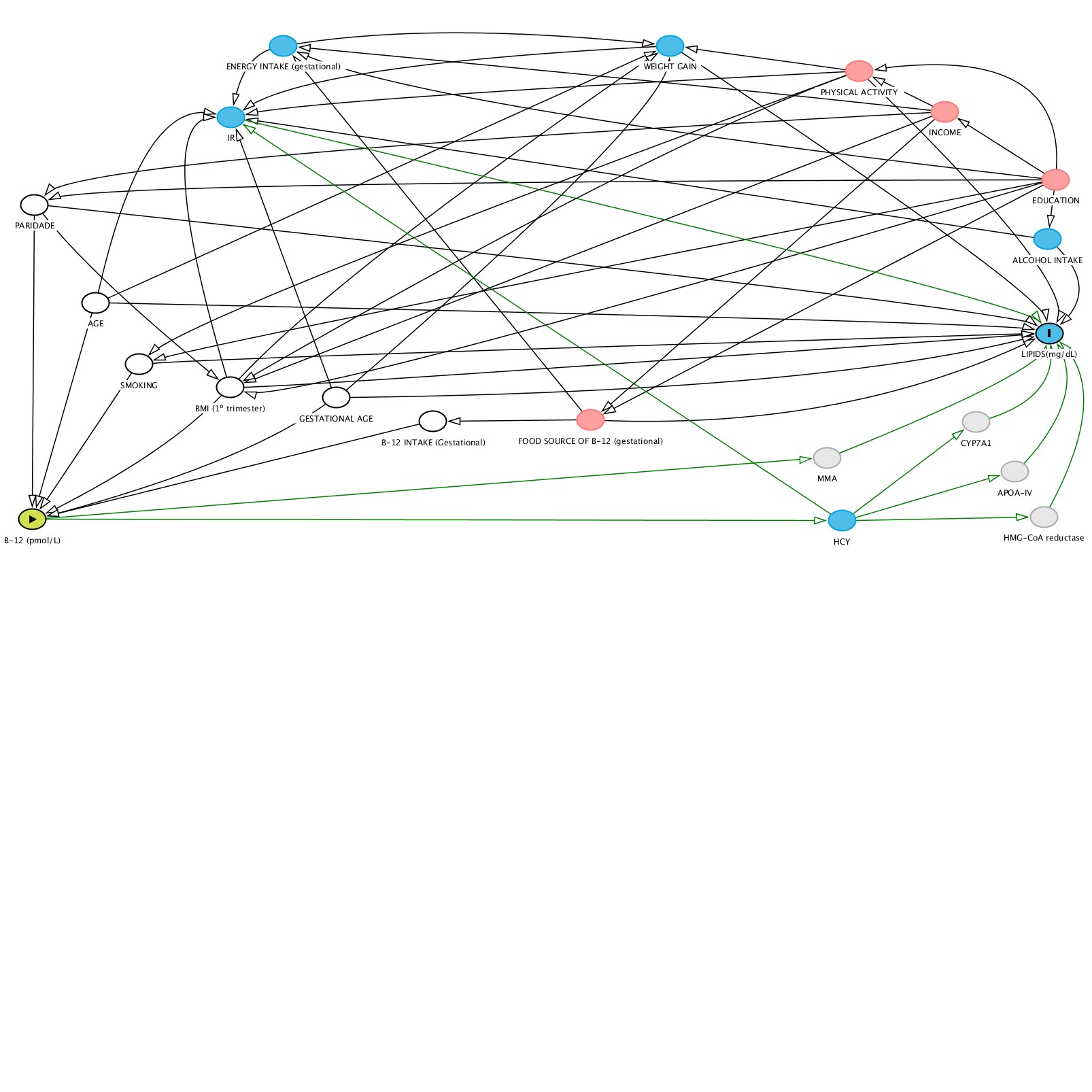
**

**Supplementary figure 2**. Acyclic Directed Graph (DAG) representing the hypothesis of relations between plasma vitamin B-12, serum lipids, and covariates.

Abbreviations: BMI, body mass index; IR, insulin resistance; CYP7A1, Cholesterol 7 alpha-hydroxylase; MTHFR, methylenetetrahydrofolate reductase; HCY, homocysteine; APOA-IV, apoprotein A-IV


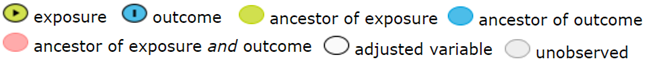


Drawn in DAGitty v 3.0

The reproducible DAG that was used to assess necessary adjustment sets (see Methods) can be found at http://dagitty.net/dags.html?id=H-5kNR

**
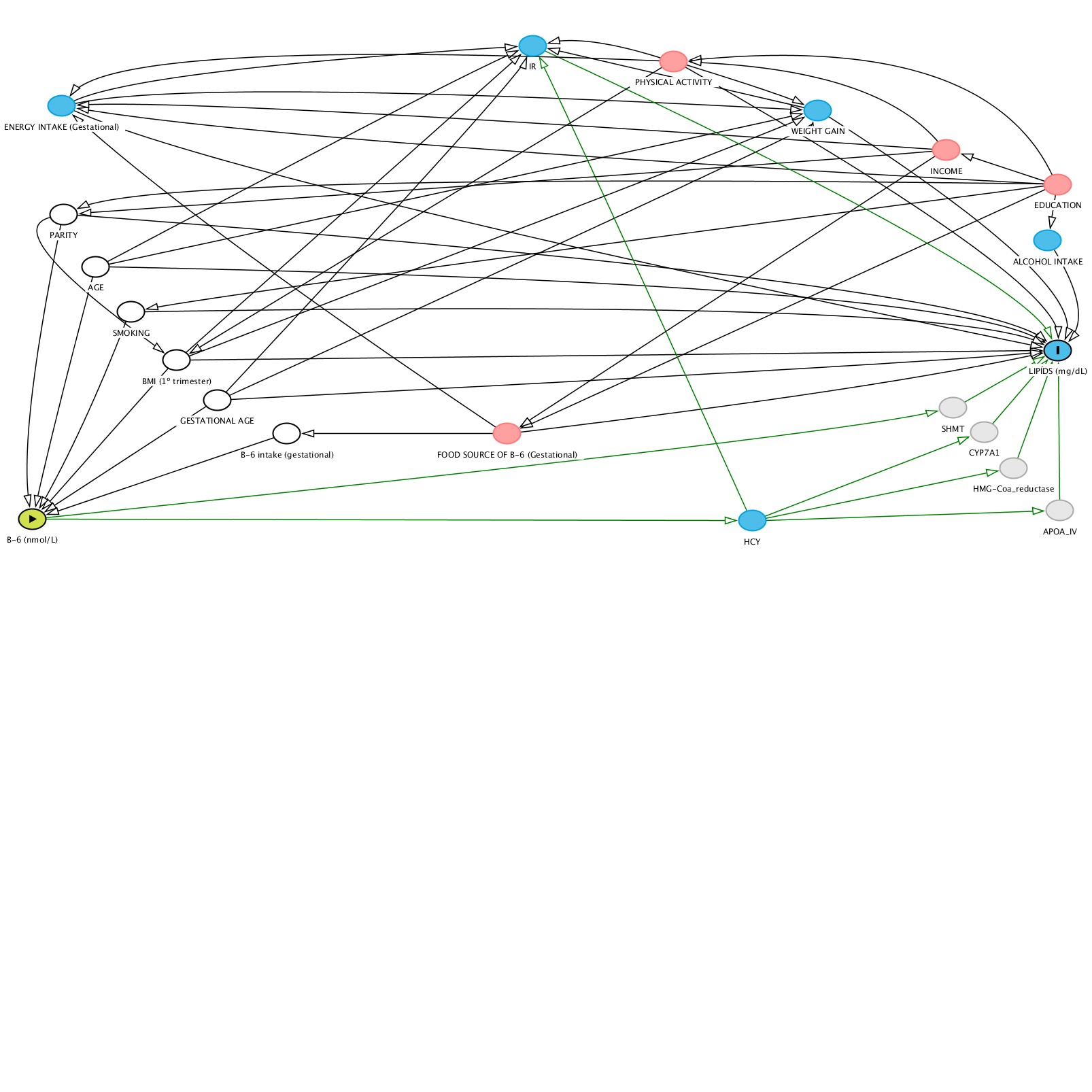
**

**Supplementary figure 3**. Acyclic Directed Graph (DAG) representing the hypothesis of relations between plasma vitamin B-6, serum lipids, and covariates.

Abbreviations: BMI, body mass index; IR, insulin resistance; CYP7A1, Cholesterol 7 alpha-hydroxylase; MTHFR, methylenetetrahydrofolate reductase; HCY, homocysteine; APOA-IV, apoprotein A-IV; SHMT, serine hydroxymethyltransferase


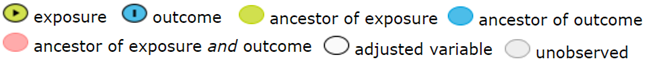


Drawn in DAGitty v 3.0

The reproducible DAG that was used to assess necessary adjustment sets (see Methods) can be found at http://dagitty.net/dags.html?id=Yh9OMU
